# Interferon-α Up-Regulates the Expression of PD-L1 Molecules on Immune Cells Through STAT3 and p38 Signaling

**DOI:** 10.3389/fimmu.2018.02129

**Published:** 2018-09-27

**Authors:** Alexandr V. Bazhin, Katharina von Ahn, Jasmin Fritz, Jens Werner, Svetlana Karakhanova

**Affiliations:** ^1^Department of General, Visceral, and Transplant Surgery, Ludwig-Maximilians-University Munich, Munich, Germany; ^2^German Cancer Consortium (DKTK), Partner Site Munich, Munich, Germany; ^3^Department of General, Visceral and Transplantation Surgery, University of Heidelberg, Heidelberg, Germany; ^4^Section Surgical Research, University of Heidelberg, Heidelberg, Germany

**Keywords:** PD-L1 (B7-H1), immunosuppression, IFNα, cancer immunotherapy, dendritic cell, STAT3 signaling

## Abstract

Interferon-α (IFNα) has one of the longest histories of use amongst cytokines in clinical oncology and has been applied for the treatment of many types of cancers. Due to its immune-activating properties, IFNα is also an attractive candidate for combinatory anti-cancer therapies. Despite its extensive use in animal tumor models as well as in several clinical trials, the different mechanisms underlying patient responses and affecting desirable clinical benefits are still under investigation. Here we show that in addition to its immune-activating properties, IFNα induces the expression of a key negative regulator, immunosuppressive PD-L1 molecule, in the majority of the specific immune cell populations, particularly in the dendritic cells (DC). DC can modulate immune responses by a variety of mechanisms, including expression of T-cell regulatory molecules and cytokines. Our results showed that treatment of DC with IFNα-2b led to pronounced up-regulation of surface expression of PD-L1 molecules, increased IL-6 and decreased IL-12 production. Moreover, we present evidence that IFNα-treated DC exhibited a reduced capacity to stimulate interferon-γ production in T cells compared to control DC. This T-cell response after treatment of DC with IFNα was recovered by a pre-treatment with an anti-PD-L1 blocking antibody. Further analyses revealed that IFNα regulated PD-L1 expression through the STAT3 and p38 signaling pathways, since blocking of STAT3 and p38 activation with specific inhibitors prevented PD-L1 up-regulation. Our findings underline the important roles of p38 and STAT3 in the regulation of PD-L1 expression and prove that IFNα induces STAT3/p38-mediated expression of PD-L1 and thereby a reduced stimulatory ability of DC. The augmentation of PD-L1 expression in immune cells through IFNα treatment should be considered by use of IFNα in an anti-cancer therapy.

## Introduction

The cytokine interferon-α (IFNα) has been used for a long time for treatment of many types of cancers, such as renal cell carcinoma, malignant melanoma or chronic myeloid leukemia (1, 2). However, the molecular mechanisms affecting patient responses and clinical outcome of IFNα therapy are still under investigation. IFNα is a member of the type I interferon family and is produced by various cell types, including monocytes, macrophages, lymphoblastoid cells, fibroblasts and plasmacytoid dendritic cells (3). Initially utilized as an anti-viral agent and meant for use in tumors where a viral origin was suspected, IFNα initiated a variety of biological activities that warranted further investigation. Indeed, this cytokine manifests direct suppressive effects on tumor cell growth *in vitro* and *in vivo* (4) and enhances tumor recognition by the increase in MHC-1 expression. Additionally, radio- and chemo-sensitizing capacities, as well as anti-angiogenic properties, have been described for IFNα (5, 6). Furthermore, Essers and colleagues have showed that the cytokine activated dormant hematopoietic stem cells *in vivo* (7). We have confirmed this phenomenon in pancreatic cancer, where we found that IFNα exhibited the ability to activate stem cell markers (8). Meanwhile, the immunostimulatory characteristics of IFNα have gained special attention since they can affect the differentiation of DC, survival of T cells, generation of CD8^+^ memory cells, macrophage activities and activation of natural killer (NK) cells (9). A lot of tumor entities, like pancreatic cancer, are characterized by reduced immunological defense (10, 11). A combination of chemotherapy with immune stimulation could improve therapy efficacy and provide an optimal cancer treatment (12). Armed with its attributes, IFNα could be an attractive candidate for combinatory therapies. Indeed, IFNα-2b (trade name Intron-A), a well-known IFN-based therapeutic (13) that is approved for the treatment of various infectious diseases as well as for many types of cancer including leukemia, lymphoma, multiple myeloma and malignant melanoma, is also actively used in multiple clinical trials (http://www.druglib.com/druginfo/intron-a/trials/).

However, it has become increasingly clear in the last few years that certain cytokines originally described as immunostimulatory and pro-inflammatory, could also up-regulate immunosuppressive molecules. Such molecules are key elements of immune inhibitory pathways, so-called immunological checkpoints, which are crucial for maintaining self-tolerance and modulating the strength of immune responses. The most prominent of them is the PD-L1 (Programmed death-ligand 1, CD274, B7-H1)–PD-1 axis. PD-L1 is a type 1 transmembrane glycoprotein and one of two ligands for the CD28 homolog programmed death-1 receptor (PD-1) (14). The protein expression of PD-L1 can be found on immune cells as well as on non-immune endothelial and epithelial cells and can be up-regulated by different agents, such as cytokines and TLR (toll-like receptor) ligands (15–19). The PD-L1 molecule plays an important role in controlling immune reactions by inhibiting T-cell response and by influencing several other cell types. It is implicated in a number of human and mouse disorders as well as in transplant rejection and pregnancy complications (14, 20, 21). Additionally, it is responsible for the chronification of viral and bacterial infections (22). The expression of this molecule contributes as well to tumor immune evasion and correlates with a poor prognosis for the cancer patient (23–26). This makes PD-L1 and its regulation an important target for on-going investigations that aim to develop new anti-cancer treatment strategies. Interferons have been shown to be able to regulate PD-L1 expression not only on tumor (25) but as well on several non-tumor cell types: IFNγ increases PD-L1 in dermal fibroblasts (15), hepatic stellate cells (27) and DC (28, 29); the up-regulation of PD-L1 in DC by IFNβ contributes to immunomodulatory effects of this cytokine in multiple sclerosis and in lipopolysaccharide-induced immune paralysis (30, 31) and the expression of PD-L1 in hepatocytes and in myeloid cells *in vitro* can be augmented by IFNα (29, 32).

The stimulation of PD-L1 expression by IFNα could make a substantial negative contribution in patient responses and clinical outcomes of IFNα therapy through increased immunosuppression. Therefore, in this work we investigated the mechanisms of regulation of PD-L1 expression in specific immune cell populations by IFNα.

## Materials and methods

### Antibodies and reagents

Anti-mouse monoclonal antibodies directly conjugated to fluorophores against the following targets of interest were used: F4/80, PD-L1, and Foxp3 (eBioscience, Germany); CD4, CD3e, CD44, CD8a, CD62L, CD45R, CD11b, CD11c, Gr-1, NK1.1, CD25, Ly6C, and PD-1 (BD Bioscience, Germany). Fc receptor binding inhibitor (anti-mouse CD16/CD32) was purchased from eBioscience (Germany). Anti-human antibodies included anti-PD-L1-PE and purified anti-PD-L1 blocking antibody (both from eBioscience, Germany). For western blot, anti-phospho-ERK42/44, anti-phospho-p38 and anti-phospho STAT3 along with their reference antibodies, anti-ERK42/44, anti-p38, and anti-STAT3 antibody (all from Cell Signaling Technology, USA), were used. Signal transduction inhibitors: UO126 (MEK1/2 inhibitor, Cell Signaling Technology, USA), SB203580 (p38 MAPK inhibitor, Cell Signaling Technology, USA), LY294002 (PI3K inhibitor), CAS457081-03-7 (Jak inhibitor 1, Calbiochem, Germany) and Cucurbitacin/JSI-124 (STAT3 inhibitor, Calbiochem, Germany). Cytokines used included IL-4, GM-CSF, TNF-α, IL-6, IL-1β, PGE2 (Promokine, Immunotools, Strathmann, Germany) and IFNα (R&D; IntronA, Interferon alfa-2b, Schering-Plough; Germany).

### Mice

C57BL/6 mice were purchased from Charles River (Germany) and bred and maintained under specific pathogen-free conditions in the animal facility of the University of Heidelberg (IBF, Heidelberg). Animal experiments were carried out after approval by the Karlsruhe regional government council (Regierungspraesidium Karlsruhe, 35-9185.81/G-184/11). The following experimental groups have been used: (1) control group (vehicle injection) and (2) treatment group (injection of IFNα). Healthy mice were intraperitoneally injected three times within 1 week with 50 μl of IFNα (2 × 10^5^ U/ml) or an equivalent volume of vehicle control with subsequent examination of the mouse splenocytes by flow cytometry.

### Generation of human DC

Buffy coats for research purposes were provided by blood donor service BSD Mannheim. Human peripheral blood myeloid DC (mDC) were isolated from freshly-prepared PBMCs from whole blood using MACS isolation kit (Miltenyi, Germany) as described elsewhere (19), according to the manufacturer's protocol. Human monocyte-derived DC (MoDC) were generated as described previously (19). Briefly, PBMCs were prepared from whole blood by Biocoll gradient centrifugation (Biochrom AG, Germany). Isolated plastic-adherent monocytes were cultured in *X-vivo* 15 medium (Bio-Whittaker, Belgium) supplemented with 1.5% human plasma in the presence of the cytokines GM-CSF and IL-4 for 5–6 days.

### Cytokine treatment

The following cytokine concentrations were used for the treatment of DC: standard cytokine cocktail for maturation (10 ng/ml TNF-α, 1,000 U/ml IL-6, 10 ng/ml IL-1β, 1 μg/ml PGE) and 500–6,000 U IFNα for generating the dose-dependent curve. For other experiments, DC were treated with 1,000 U/ml IFNα for 24 h and subjected to FACS analysis, western blot or DC-T-cell co-cultures.

### DC-T-cell co-cultures

DC and T-cells were co-cultured as described previously (19). Generated or freshly isolated DC were pretreated with 1,000 U/ml IFNα for 24 h and then washed twice with medium. CD4^+^ cells were isolated from freshly-prepared PBMCs using MACS beads (Miltenyi, Germany). CD4^+^ cells and DC were cultured together in 96-well round-bottom plates for 5 days, after which the production of cytokines in supernatants was determined in triplicates using Luminex assay.

### Immunocytochemistry

Immunocytochemistry was performed as described elsewhere (8). Briefly, cells were immobilized on microscope slides using a cytocentrifuge, fixed with 3.7% formaldehyde, pre-absorbed and incubated with anti-PD-L1 or control antibody. After incubation with secondary biotin-coupled antibodies followed by streptavidin–phosphatase complexes, PD-L1 was detected through the formation of a colored reaction product from the hydrolysis of substrate by alkaline phosphatase. Hematoxylin was used for counterstaining nuclei.

### Western blot analysis

DC treated with 1,000 U/ml IFNα for the indicated periods of time were harvested, lysed in 2× sample buffer (100 mM Tris, pH 6.8, 4% SDS, 16% glycerol, 0.57 M β-mercaptoethanol, 0.01% bromophenol blue) and heated to 95°C for 5 min. SDS-PAGE was carried out after loading the proteins onto a 10% polyacrylamide gel. After transfer to PVDF membranes, the blots were sequentially blocked for 2 h using 5% milk in TBST solution (10 mM Tris, pH 8.0, 150 mM NaCl, 0.1% Tween-20), incubated overnight with primary antibodies in TBST containing 5% BSA, washed three times in TBST and incubated with HRP-conjugated secondary antibodies. Subsequently, the proteins of interest were detected by chemiluminescence produced by HRP-catalyzed oxidation of ECL substrate (Lumigen TMA6, UK).

### Flow cytometry analysis

Cells were collected, washed and incubated for 15 min at 4°C in FACS buffer containing antibodies directly conjugated to fluorophores. The fluorescence was evaluated using a FACSCanto II flow cytometer (BD Biosciences, Germany) and data were analyzed with Diva and FlowJo Software (BD Biosciences, Germany). All isotype controls had fluorescence values that remained below a threshold of 1 × 10^3^. For analysis of murine leukocytes, a freshly-isolated splenocyte cell suspension was prepared as described previously (33), resuspended in the stain buffer (PBS supplemented with 1% mouse serum and 1 mM EDTA), counted and adjusted to a concentration of 4 × 10^7^ cells/mL. Cells were blocked with anti-mouse CD16/CD32 antibodies at 4°C in the dark for 10 min and then incubated with stain buffer containing various combinations of previously titrated monoclonal antibodies at 4°C in the dark for 15 min. After two washing steps with the stain buffer, the cells were used for flow cytometry analysis. For intracellular staining, Foxp3 buffer set was used according to the manufacturer's instruction. All the gates were set according to the corresponding fluorescence minus one (FMO) controls. For the gating strategy see Figure S1 and the manuscript from Fritz et al. (33).

### Luminex assay

Analyses of human cytokines IL-6, IL-12(p40), IFNγ, IL-1b,−4,−5,−10,−12(p70), and−17 as well as TNF-α in culture supernatants were performed as described elsewhere (34) using a MILLIPLEX® MAP Kit (Millipore GmbH, Schwalbach/TS, Germany) according to the manufacturer's instructions. The measurements were performed in triplicates using a Luminex® 100/200 System.

### Statistical analysis

The data were analyzed with unpaired Student's *t*-test or one-way ANOVA using GraphPad PRISM 5.0. Values with *p* < 0.05 were considered significant. Quantitative data are expressed as mean ± SEM.

## Results

### Expression of PD-L1 and PD-1 on various populations of murine immune cells

Our first aim was to determine the distribution of PD-L1 and PD-1 expression on extracellular surfaces of different specific immune cell populations. For this purpose, splenocytes from BL6 mice were subjected to a deep FACS analyses (Figure S1) to obtain the results summarized in Table 1. Expression of PD-L1 was found on all immune cells tested with higher percentages of PD-L1^+^ cells (more than 80%) in conventional myeloid DC (cDC, CD11c^high^CD11b^+^), macrophage (CD11b^+^Gr-1^−^F4/80^+^), naïve CD8 cell (CD62L^+^CD44^−^), effector memory (em) CD4 cell (CD62L^−^CD44^+^) and regulatory T cell (Treg, CD4^+^CD25^+^FoxP3^+^) populations. The lowest percentages of PD-L1^+^ cells (lower than 5% positive cells) were found in em cells (CD62L^−^CD44^+^) and naïve CD8^+^ cells as well as in activated conventional (con) T cells (Tcon, CD4^+^CD25^+^FoxP3^−^). Expression of PD-1 was also found on all lymphoid cell populations analyzed (Table 1). Thus, PD-L1 and its receptor are commonly present on various murine immune cells.

**Table 1:** Distribution of PD-1 and PD-L1 expression on immune cell subpopulations.

	**PD-1**		**PD-L1**	
	**Cell frequency, %**	**MFI**	**Cell frequency, %**	**MFI**
CD3^−^NK1.1^+^CD4^−^	4.02 (±1.35)	992.6 (±407.4)	n.d. (not detected)	n.d.
CD3^+^NK1.1^+^CD4^+^	22.55 (±12.98)	2,308 (±298.2)	n.d.	n.d.
CD11c^high^CD11b^+^	n.d.	n.d.	91.73 (±4.44)	22,500 (±25.5)
CD11c^int^CD45R^+^	n.d.	n.d.	58.3 (±14.79)	5,913 (±233.3)
CD11b^+^Gr-1^−^F4/80^+^	n.d.	n.d.	82.3 (±0.63)	9,223 (±70)
CD11b^+^Gr-1^+^	n.d.	n.d.	21.58 (±11.69)	7,669 (±2,250)
CD11b^+^Gr-1^high^Ly-6C^int^	n.d.	n.d.	15.27 (±6.99)	4,040 (±2,329)
CD11b^+^Gr-1^int^Ly-6C^high^	n.d.	n.d.	44 (±10.52)	6,772 (±1,539)
CD8^+^CD3^+^	11.16 (±6.72)	1,103 (±437.7)	81 (±26.73)	683 (±49.5)
CD62L^−^CD44^+^CD8^+^	n.d.	n.d.	1.34 (±0.26)	993 (±162.6)
CD62L^+^CD44^+^CD8^+^	n.d.	n.d.	64.65 (±0.26)	876 (±86.3)
CD62L^+^CD44^−^CD8^+^	n.d.	n.d.	83.35 (±0.26)	554 (±22.6)
CD62L^−^CD44^−^CD8^+^	n.d.	n.d.	1.47 (±0.26)	671 (±166.9)
CD4^+^CD3^+^	14.54 (±4.88)	1,468 (±429.3)	57.87 (±27.65)	1,568 (±1,686)
CD62L^−^CD44^+^CD4^+^	n.d.	n.d.	85.8 (±6.79)	1,490 (±16.3)
CD62L^+^CD44^+^CD4^+^	n.d.	n.d.	44.05 (±7.99)	1,688 (±359.9)
CD62L^+^CD44^−^CD4^+^	n.d.	n.d.	48.75 (±40.94)	801 (±14.1)
CD62L^−^CD44^−^CD4^+^	n.d.	n.d.	41.9 (±40.94)	1,494 (±304.1)
CD25^dim^FoxP3^+^CD4^+^	39.18 (±9.83)	2,226 (±343.5)	54.3 (±40.94)	3,156 (±2,771)
CD25^+^FoxP3^+^CD4^+^	24.15 (±6.65)	2,213 (±355.8)	84.45 (±9.12)	3,223 (±2,785)
CD25^+^FoxP3^−^CD4^+^	18.68 (±7.59)	1,654 (±266.4)	4.2 (±5.06)	2,632 (±2,722)
CD25^−^FoxP3^−^CD4^+^	77.07 (±2.74)	1,400 (±187.3)	70.25 (±0.64)	2,583 (±2,786)

### IFNα up-regulates *ex vivo* and *in vivo* expression of PD-L1 on mouse leukocytes

**Figure 1:**
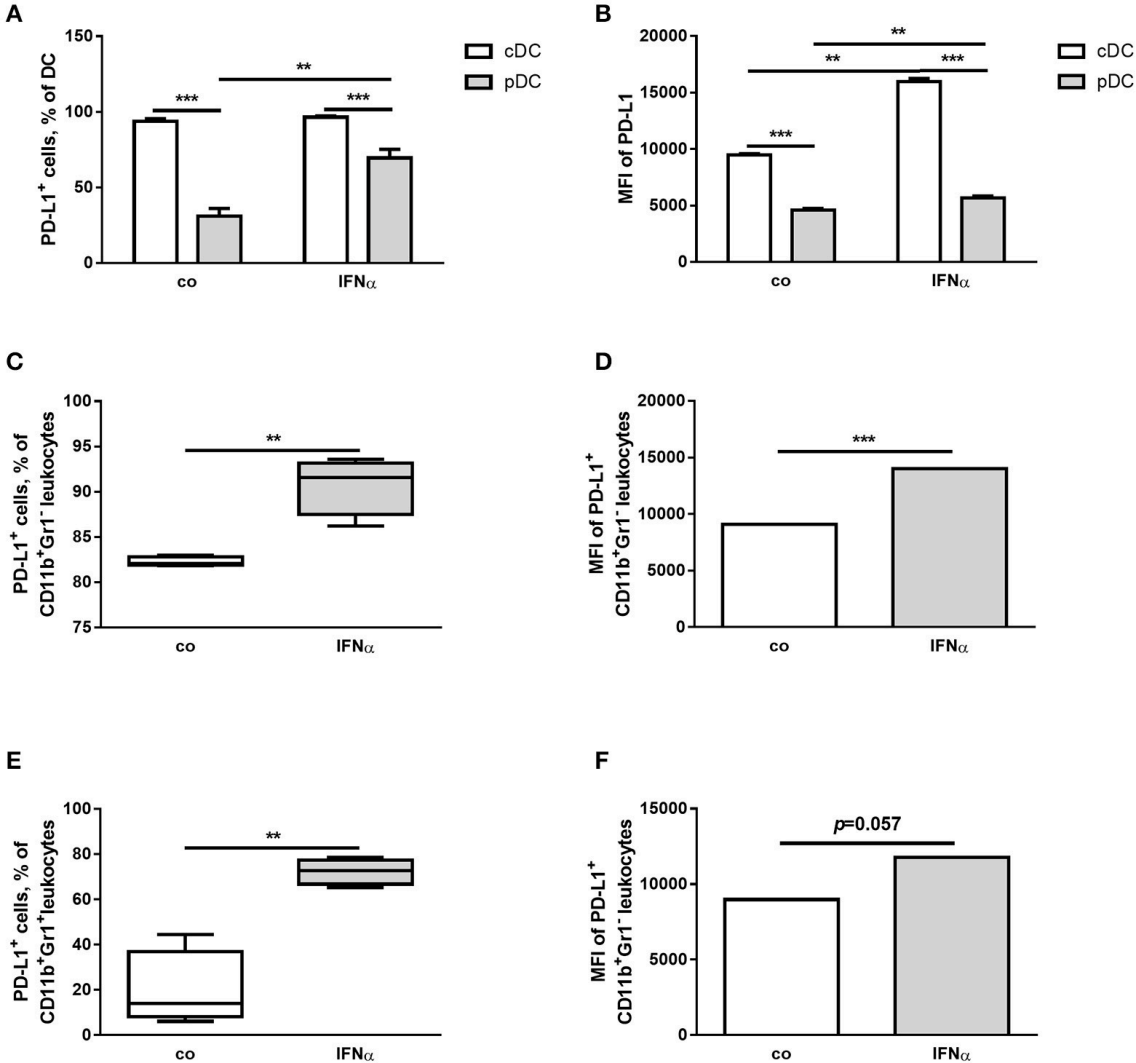
IFNα up-regulates PD-L1 expression on different myeloid immune cell populations *ex vivo*. FACS analysis of PD-L1 expression on the surface of different myeloid immune cells from splenocytes of healthy mice. Splenocytes were isolated, treated for 24 h with 1,000 U/ml IFNα and investigated by flow cytometry. The results are presented as interleaved bars **(A,B)**, as box and whiskers plots **(C,E)** or column bar graphs **(D,F)** and statistically analyzed using two-way ANOVA **(A,B)** or unpaired *T*-tests **(C–F)**, *n* = 4, ^**^p < 0.01, and ^***^p < 0.001.

In the next step, isolated splenocytes were treated for 24 h with 1,000 U/ml IFNα and PD-L1 expression on splenocytes was detected with flow cytometry. IFNα treatment led to increased percentages of cells expressing PD-L1 in plasmacytoid DC (pDC), macrophage and CD11b^+^Gr^−^1^+^ cell populations, but not in cDC in the myeloid leukocyte population (Figure 1). The reason is that cDC are already PD-L1 positive to a high percentage (Table 1). However, the level of PD-L1 expression (measured in MFI, mean fluorescent intensity) was positively affected in both DC subpopulations of CD11b^+^ cell (Figure 1).

**Figure 2:**
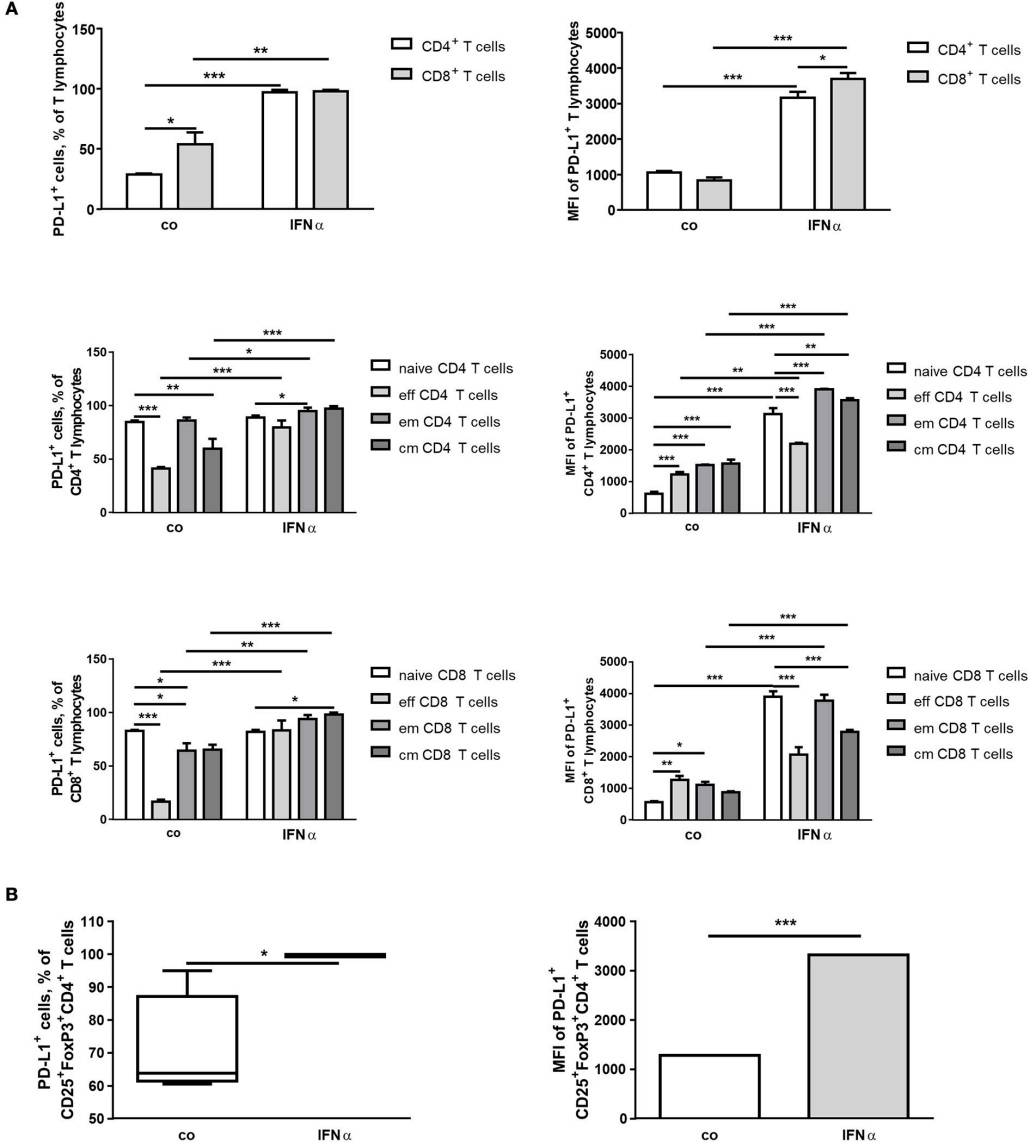
IFNα up-regulates PD-L1 expression on different T-cell populations *ex vivo*. FACS analysis of PD-L1 expression on the surface of different lymphoid immune cells from spleens of healthy mice. Splenocytes were isolated, treated for 24 h with 1,000 U/ml IFNα and investigated by flow cytometry. The results are presented as interleaved bars **(A)** or as box and whiskers plot or column bar graph **(B)** and statistically analyzed using two-way ANOVA or unpaired *T*-tests, *n* = 4, ^*^p < 0.05, ^**^p < 0.01, and ^***^p < 0.001.

In the lymphocyte population, IFNα treatment increased a number of PD-L1^+^ cells and up-regulated the expression of PD-L1 in both CD4^+^ and CD8^+^ cells (Figure 2). In specific subpopulations of CD4^+^ cells, the percentage of cells expressing PD-L1^+^ was increased in eff, em and cm (CD62L^+^CD44^+^) cells and the level of PD-L1 expression was higher after IFNα treatment in all subpopulations (Figure 2). Interferon-α was observed to exert a similar effect in Treg (Figure 2). For the gated CD8^+^ cells, an increased number of PD-L1^+^ cells was found in eff, em and cm populations (Figure 2). The level of PD-L1 expression measured in MFI was higher after treatment with IFNα in all subpopulations except eff CD8^+^ cells (Figure 2). Thus, IFNα is capable of up-regulating PD-L1 expression in different murine immune cells.

**Figure 3:**
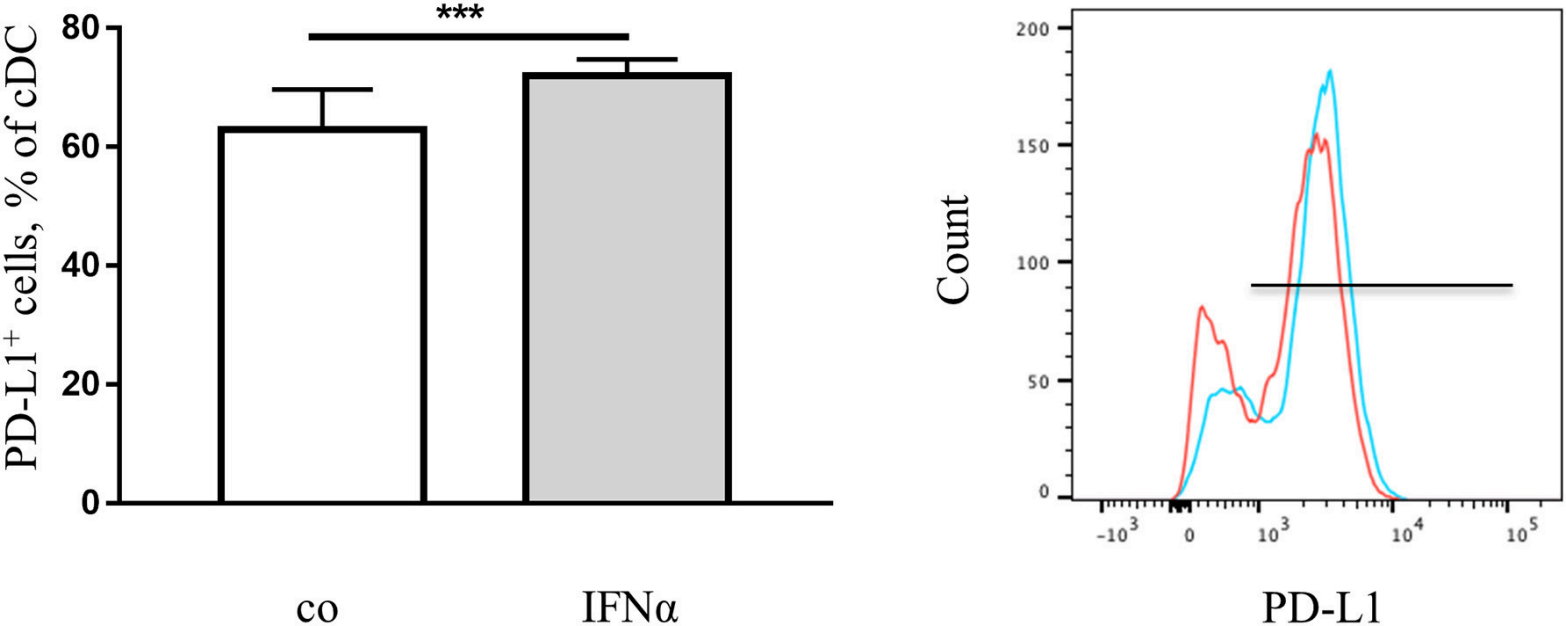
IFNα up-regulates PD-L1 expression on DC *in vivo*. FACS analysis of PD-L1 expression on the surface of DC from spleens of healthy mice with or without treatment with IFNα. Splenocytes were isolated and examined using flow cytometry. The results are presented as percent of positive cells in a column bar graph and statistically analyzed using unpaired *T*-test, *n* = 10, ^***^p < 0.001. A representative FACS histogram (red-control, blue-IFNα) is shown.

Since the highest initial percentage of PD-L1^+^ cells and up-regulation of PD-L1 after IFNα treatment was observed in DC in *ex vivo* splenocytes cultures, the *in vivo* regulation of PD-L1 expression by IFNα was examined and we verified a similar up-regulation in mouse DC (Figure 3) as well as in other myeloid cells (data not shown).

### IFNα up-regulates the expression of PD-L1, increases the production of IL-6 and decreases the production of IL-12 by human DC

**Figure 4:**
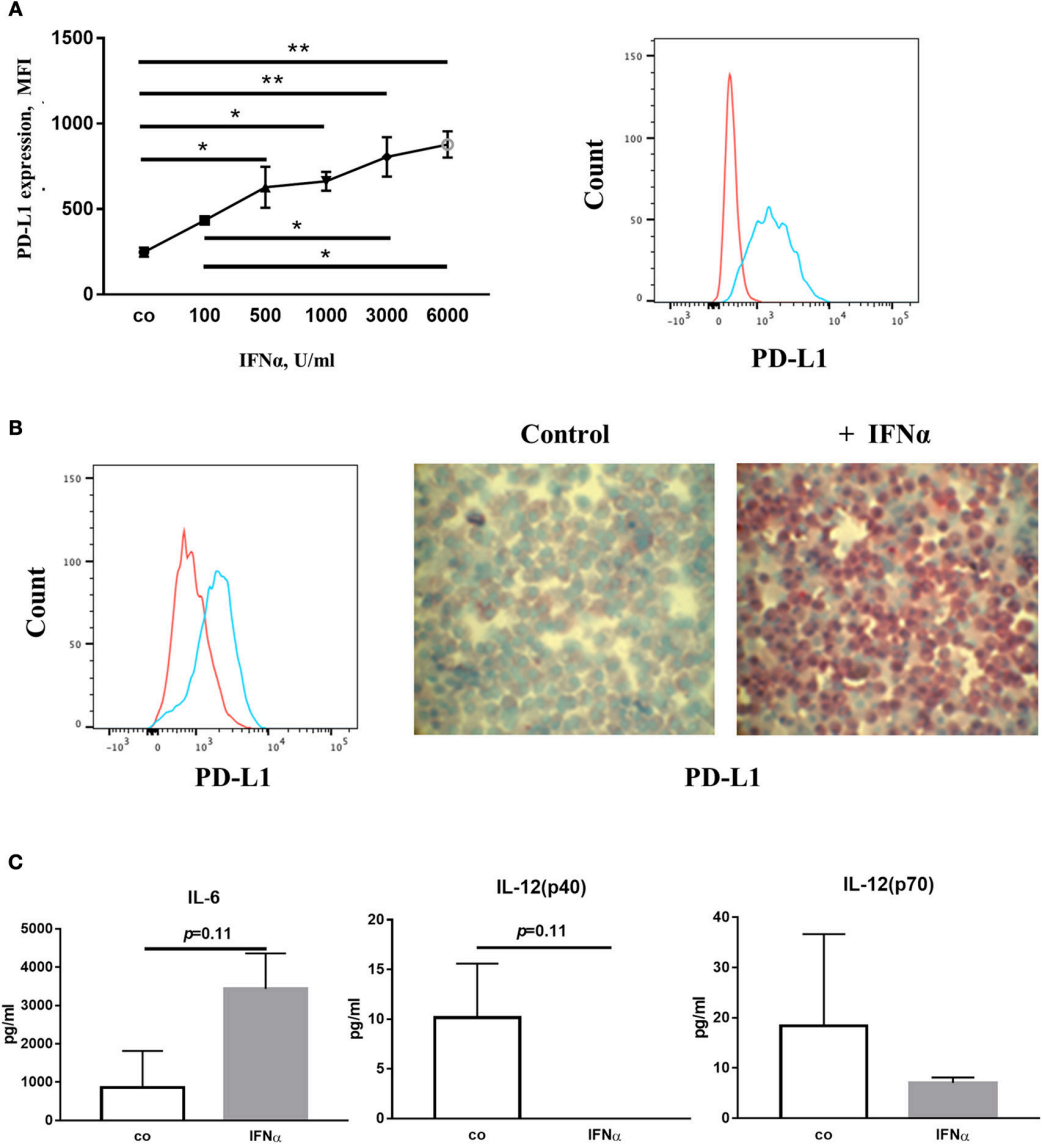
IFNα up-regulates PD-L1 expression on human dendritic cells. **(A)** FACS analysis of PD-L1 expression on the surface of mDC. Myeloid DC were isolated, treated for 24 h with IFNα and analyzed with flow cytometry. The results are presented as column mean, error bars and mean connected and statistically analyzed using ordinary one-way ANOVA with the Tukey's multiple comparisons post-test, *n* = 2, ^*^p < 0.05 and ^**^p < 0.01. **(B)** FACS analysis and Immunocytochemistry (ICH) of PD-L1 expression on the surface of MoDC. MoDC were treated for 24 h with 1,000 U/ml IFNα and analyzed with flow cytometry. Alternatively, cytospin slides were produced and ICH was performed using an anti-PD-L1 primary antibody followed by a biotinylated secondary antibody. FACS histograms (red-control, blue-IFNα) and ICH pictures are representative for independent experiments. **(C)** Cytokines in the supernatant of the IFNα-treated DC analyzed with Luminex assays. The results are presented as column bar graphs and statistically analyzed using unpaired *T*-test, *n* = 2.

To increase the clinical relevance of our study, we extended our research to investigate PD-L1 regulation in human DC facilitated through the use of IFN-based therapeutic IFNα-2b (Intron A). Myeloid DC were isolated from buffy coats of healthy human subjects and cultivated with different concentrations of IFNα as indicated in Figure 4. The treatment induced dose-dependent up-regulation of PD-L1 expression in the cells (Figure 4A). Similarly, PD-L1 expression was up-regulated in MoDC obtained from buffy coats of human healthy donors (Figure 4B). Remarkably, an increased PD-L1 expression was still induced when mDC and MoDC were pulsed with IFNα after incubation with a classical maturation cytokine cocktail (Figure S2). Therefore, IFNα can specifically induce PD-L1 overexpression in human DC.

Produced cytokines are important indicators of DC functionality. After the treatment of DC with IFNα, 10 human cytokines were measured in cell culture supernatants using the Luminex platform. We found an increased concentration of IL-6 and a decreased concentration of IL-12(p40) and IL-12(p70) in the supernatants (Figure 4C) after IFNα treatment. However, the production of IFNγ, IL-1β,−4,−5,−10, and−17 as well as TNF-α was not affected (data not shown).

### Blocking PD-L1 leads to recovery of IFNγ production by CD4^+^ lymphocytes cultivated with IFNα-treated mDC

**Figure 5:**
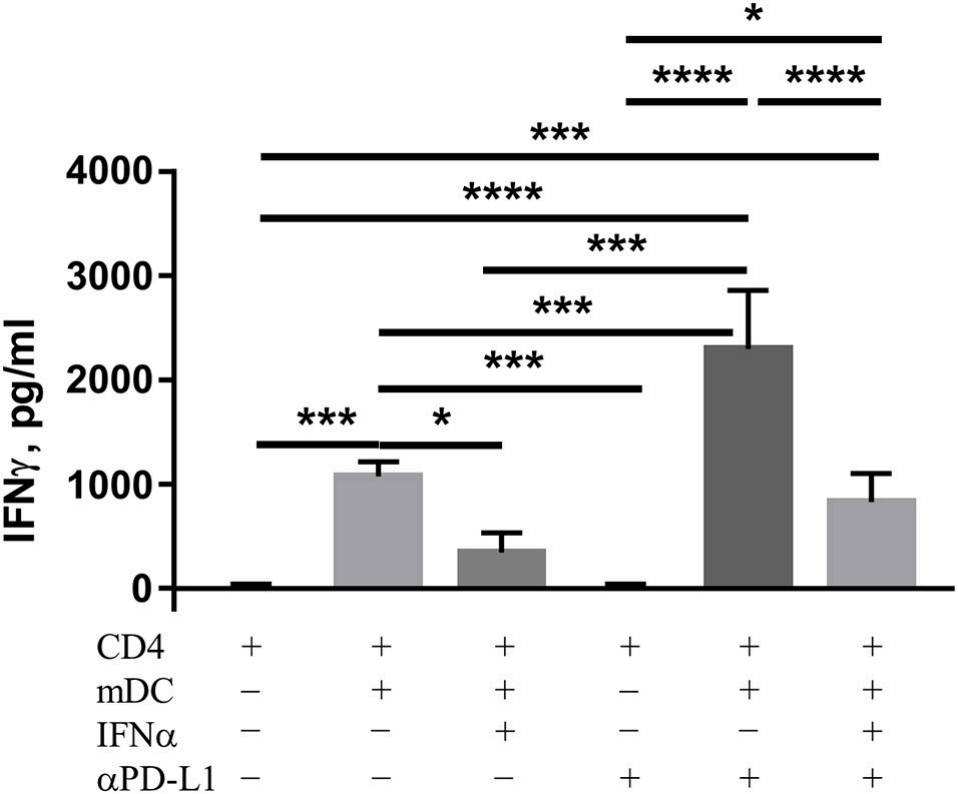
PD-L1 controls IFNγ release from CD4 T cells stimulated by DC. Allogeneic CD4 cells were isolated and cocultured with DC in the presence or the absence of anti-PD-L1 blocking antibody or vehicle control. The level of IFNγ in supernatants was determined by Luminex assay. The results are presented as column bar graphs and statistically analyzed using ordinary one-way ANOVA with the Tukey's multiple comparisons post-test, *n* = 3–6, ^*^p < 0.05, ^***^p < 0.001, and ^****^p < 0.0001.

The expression of PD-L1 on DC negatively modulates their ability to activate CD4^+^ lymphocytes and subsequent IFNγ production (19). Based on our findings above indicating that IFNα treatment affects IFNγ production by co-cultured CD4^+^ lymphocytes but not DC, we aimed to investigate whether the blocking of PD-L1 protein with antibodies [anti (α)-PD-L1] could influence IFNγ production in our co-culture experiments. In order to achieve the above aim, we co-cultivated CD4^+^ cells with mDC pretreated with IFNα, anti-PD-L1 or both IFNα and anti-PD-L1. As expected, the co-cultivation of CD4^+^ cells with untreated mDC led to an increase in the amount of IFNγ in the supernatants and addition of anti-PD-L1 antibody further improved the cytokine release (Figure 5). Treatment of CD4^+^ cells with anti-PD-L1 but without mDC did not induce IFNγ production. Pre-incubation of mDC with IFNα resulted in a decrease in the amount of IFNγ in the supernatants of co-cultivated CD4^+^ cells (Figure 5), which could be explained by the up-regulation of PD-L1 expression on mDC. Indeed, blocking of IFNα-induced PD-L1 on DC led to higher IFNγ production from co-cultivated CD4^+^ cells compared to the co-cultures containing IFNα-DC without an αPD-L1 antibody blockade. The restoration of IFNγ production, resulted in the same amount of the cytokine released into the supernatant as in the samples that were co-cultivated with mDC without any pre-treatment (Figure 5). Thus, blocking PD-L1 leads to the recovery of IFNγ production by CD4^+^ lymphocytes activated with IFNα-treated mDC. The same results were observed when MoDC were used instead of mDC (data not shown).

### Inhibitors of STAT3, p38 and jak down-regulate the expression of PD-L1 induced by IFNα, whilst PI3K or ERK inhibitors do not exert this effect

**Figure 6:**
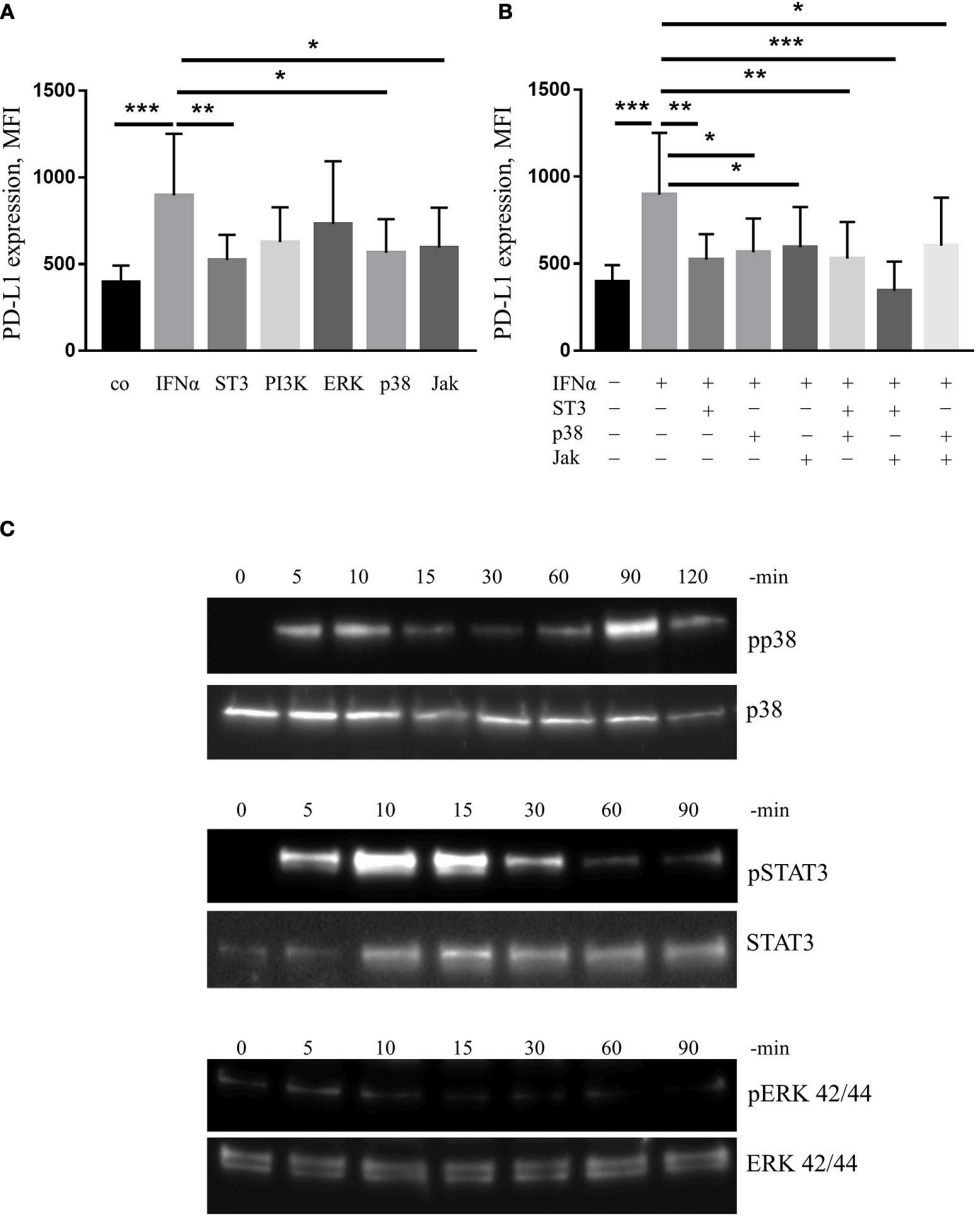
IFNα up-regulates PD-L1 expression in mDC in p38/STAT3-dependent manner. **(A,B)** FACS analysis of PD-L1 expression on mDC activated for 24 h with 1,000 U/ml IFNα with or without 1 h pre-incubation with signal transduction inhibitors of p38, STAT3 (ST3), PI3K, ERK, p38, and Jak. The results are presented as column bar graphs and statistically analyzed using ordinary one-way ANOVA with the Dunnett's multiple comparisons post-test, *n* = 8–10, ^*^p < 0.05, ^**^p < 0.01, and ^***^p < 0.001. **(C)** Western blot analysis of p38, STAT3, and ERK phosphorylation (pp38, pERK, and pSTAT3 for phosphorylated form) in mDC before and after activation for indicated period of time with IFNα.

Finally, we wanted to identify the molecular mechanisms by which IFNα up-regulates PD-L1 expression. We treated human mDC obtained from buffy coats with inhibitors of different signaling proteins as indicated in Figure 6. We found that IFNα-induced PD-L1 expression was down-regulated by inhibitors of p38, Jak and STAT3 but not by inhibitors of PI3K or ERK (Figure 6A). However, combinations of two different inhibitors did not show additive inhibitory effects (Figure 6B). In line with these observations, IFNα could increase the phosphorylation of p38 and STAT3 as shown by western blot (Figure 6C). In addition, we did not observe any effect on PD-L1 expression when NF-kB or STAT1/STAT5 were blocked with specific inhibitors (data not shown). Therefore, we concluded that signaling cascades including Jak/STAT3 and p38 could be involved in the regulation of PD-L1 expression.

## Discussion

In this study, we showed for the first time that PD-L1 molecule was expressed and could be up-regulated in the majority of specific immune cell populations by IFNα.

Programmed death-ligand 1, expressed on different immune cells, such as MDSC (35, 36), T cells (37), DC (19, 38), macrophages (39), pDC (40), is able to cause immunosuppression. Taking into account the ubiquitous expression of PD-L1 receptor (PD-1) on non-myeloid specific immune cells (CD8, CD4 and Treg), our results indicate that immunotherapy with IFNα could lead to an undesirable side effect of general immunosuppression and consequently to increased tumor immune evasion or chronification of infection. Our data could also explain the insufficiency of IFNα therapy observed in several models, despite its promising *in vitro* and immunomonitoring results (41–44). We propose that the combination of IFNα with checkpoint inhibitors like PD-L1 blocking antibody, could repress this immunosuppressive path and improve the efficiency of IFNα therapy by uncoiling the immune costimulatory potential of IFNα. While the majority of the DC are already positive for PD-L1, the intensity of this expression could be strongly up-regulated in our study by IFNα *in vivo, ex vivo*, and *in vitro*. DC play a crucial role in the control of adaptive tolerance and immunity and modulate immune responses by multiple mechanisms, including the production of cytokines and expression of T-cell regulatory molecules. Their final stimulatory capacity depends on the balance between stimulatory and suppressive pathways, whereby PD-L1 provides one of the most decisive suppressive signals and leads to unfavorable outcomes due to decreased anti-tumor immunity. This becomes particularly evident by documentation of reduced DC functions in several types of cancer and chronic infections (45–53). In cases utilizing vaccination as immunotherapeutic approaches against tumor and viral antigens, it is important that the produced DC display their full activation capacity (54, 55). Therefore, many therapies aim to activate DC to increase their stimulatory potential. IFNα was suggested as one of potential candidates for non-specific immune stimulation or as a replacement of IL-4 in the process of Mo-DC production (56–59). In accordance with our data, the MoDC produced from the monocytes treated with GM-CSF and IFNα (IFNα/GM-CSF MoDC) had a higher expression of PD-L1 molecules compared to those treated with IL-4/GM-CSF (60). In this case, the therapeutic efficacy of DC produced or activated with IFNα could be improved by the simultaneous reduction of PD-L1 expression.

The IFNα-treated DC demonstrated increased IL-6 and decreased IL-12 production. This is in line with the observation that TLR-antigen presenting cells, which express a high amount of PD-L1 and fail to induce T-cell proliferation, also exhibited increased IL-6 production (61). IFNα can inhibit IL-12 production in mouse splenocytes (62) and IL-12p40 production in human MoDC (57, 63). However, IL-6 has been shown to down-regulate IL-12 production by human MoDC (64). In our model, the cause of decreased IL-12 production, whether directly through IFNα treatment or partially caused as an effect of IL-6, remains to be clarified. Moreover, we reported previously that IL-6 is able to induce PD-L1 in DC *per se* (18), thus PD-L1 expression could be boosted additionally by this loop.

In accordance with the findings of other researchers, we have shown previously that specific up-regulation of PD-L1 regulatory molecules on DC surfaces affect the capacity of DC to induce T-cell cytokine production (19, 30). In line with these previous results, we found that IFNα-treated DC strongly down-regulated IFNγ production by T cells. However, IL-12, produced by antigen-presenting cells including DC, can also control the production of IFNγ by T cells (65) and this is as well-evident when IFNγ release by co-cultured CD4 T cells decreased in response to diminished IL-12p70 production by IL-6 treated DC (64). In our study, the decrease in IL-12 production might not have an important role in stimulating IFNγ release in the co-cultures, since blocking PD-L1 on IFNα-treated DC could almost completely restore IFNγ release. Thus, in our experimental settings, the inhibitory ability of IFNα is directly linked to increased PD-L1 expression on DC. Given that IFNγ induces the expression of PD-L1 in DC (28, 29), repression of IFNγ production by T cells by IFNα could negatively regulate de novo expression of PD-L1 on the surface of DC during immune response *in vivo*.

The decisive role held by PD-L1 molecule in controlling immune response and anti-tumor immunity urges the investigation of signaling events controlling its up-regulation. Elucidation of the PD-L1 regulation on DC is an emerging field, as these cells govern the decision between tolerance and immunity. By canonical way, ligand engagement of the IFNα receptor (IFNAR, composed of the IFNAR1 and IFNAR2 subunits) activates Jak1 and tyrosine kinase 2 (TYK2) and can result in the recruitment of STATs as well as MAPKs, PI3K, Akt, NF-kB and PRMT1 (66). There are three predominant STAT complexes that might be formed in response to IFNs: (i) The interferon-stimulated gene factor 3 (ISGF3) complex [STAT1/STAT2 and IFN-regulatory factor 9 (IRF9)], which binds to IFN-stimulated response element (ISRE) sequences and activate antiviral genes, (ii) STAT1 homodimers, which bind to gamma-activated sequences (GASs) and initiate pro-inflammatory genes, and (iii) STAT3 homodimers which indirectly suppress pro-inflammatory gene expression. Our experiments revealed that IFNα regulates PD-L1 expression in a Jak-, STAT3-, and p38-dependent manner. Whether other downstream effectors of IFN1 signaling might be involved in the modulation of PD-L1 expression needs further investigations. Similarly, in mouse IL-27-treated pDC, STAT3-dependent enhancement of PD-L1 was described (40). The TLR-agonist-induced PD-L1 expression was modulated in a MAPK/STAT3-dependent way, whereby STAT3 was rapidly recruited to the PD-L1 promoter and in agreement with our findings, blocking of STAT3 activation prevented PD-L1 expression (61). We showed previously that IL-27-induced specific up-regulation of the PD-L1 regulatory molecule on DC was accompanied by the phosphorylation of another STAT family member, STAT1 protein (19), but we did not observe STAT1 involvement in the IFNα-induced PD-L1 expression.

Furthermore, in several tumor cell types, the role of Jaks/STATs in PD-L1 regulation was highlighted recently. Attenuation of IFNγ-induced PD-L1 expression in melanoma cells was proven to happen via down-regulation of the Jak/STAT/IRF-1 signaling pathway (67, 68), while activation of Jaks led to PD-L1 up-regulation in hematopoietic tumor cell lines and primary tumor cells (69). In contrast to our previous observations, neither PI3K nor ERK activation was essential for IFNα-induced PD-L1 expression (18). Thus, our findings underline the important roles of STATs in the regulation of PD-L1 expression and are in agreement with Barton et al. who states that the stimulatory ability of APCs depends on the degree of STAT3 activation (70). We can also speculate that therapies that target p38 and STAT3 pathways could potentially produce a desirable secondary effect on PD-L1 expression.

Another type I IFN, IFNβ, is also used as an immunomodulatory cytokine in the treatment of multiple sclerosis (71) and in anti-cancer therapies, for example against nasopharyngeal carcinoma (72). Since other type I IFNs bind the same IFNR1/2 receptor complexes and have a similar mode of action (73), one could assume that the effects found in our IFNα study might be valid for them as well and so more attention needs to be paid to these IFNs in future studies. Giving further evidence to support this hypothesis, IFNβ-dependent facilitated increase in PD-L1 expression in DC was documented in multiple sclerosis and in immune paralysis (30, 31).

Our findings underline the important roles of p38 and STAT3 in the regulation of PD-L1 expression and showed that IFNα-2b, which is clinically used for a wide range of indications including cancers, induced STAT3/p38-mediated expression of PD-L1, favoring a reduction in the stimulatory ability of DC. Particular consideration should be given to the enhanced PD-L1 expression in multiple immune cell types caused by the use of IFNα in anti-cancer therapy in the future.

The obtained results reveal a new avenue for the development of novel and optimization of existing therapeutic strategies with IFNα, in order to precisely modulate PD-L1 expression in DC and other target cells.

## Author contributions

SK and AB participated in the research design; All authors participated in carrying out the research and analyzing the data; AB and SK participated in writing the manuscript and critical correction of the manuscript. AB, JW, and SK administered this work. All authors discussed the results and implications and gave constructive feedback on the manuscript at all stages.

### Conflict of interest statement

The authors declare that the research was conducted in the absence of any commercial or financial relationships that could be construed as a potential conflict of interest.
