# Supplementary figures and images for: Interferon-α Up-Regulates the Expression of PD-L1 Molecules on Immune Cells Through STAT3 and p38 Signaling
Source: Front Immunol. 2018 Sep 27;9:2129. doi: 10.3389/fimmu.2018.02129 (PMC6190899; doi:10.3389/fimmu.2018.02129)

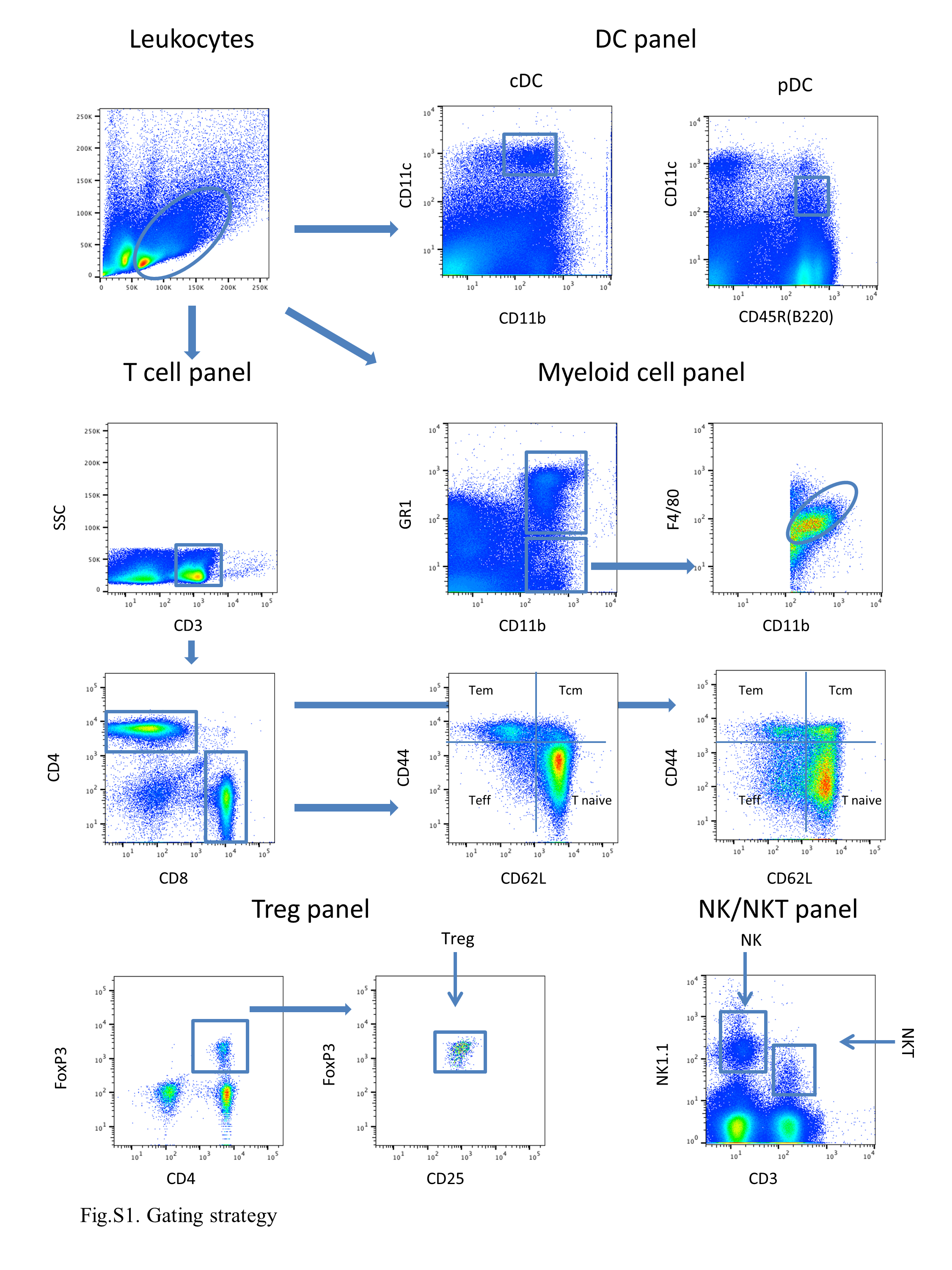

Supplement: Supplementary file 1 [file Image_1.tif]

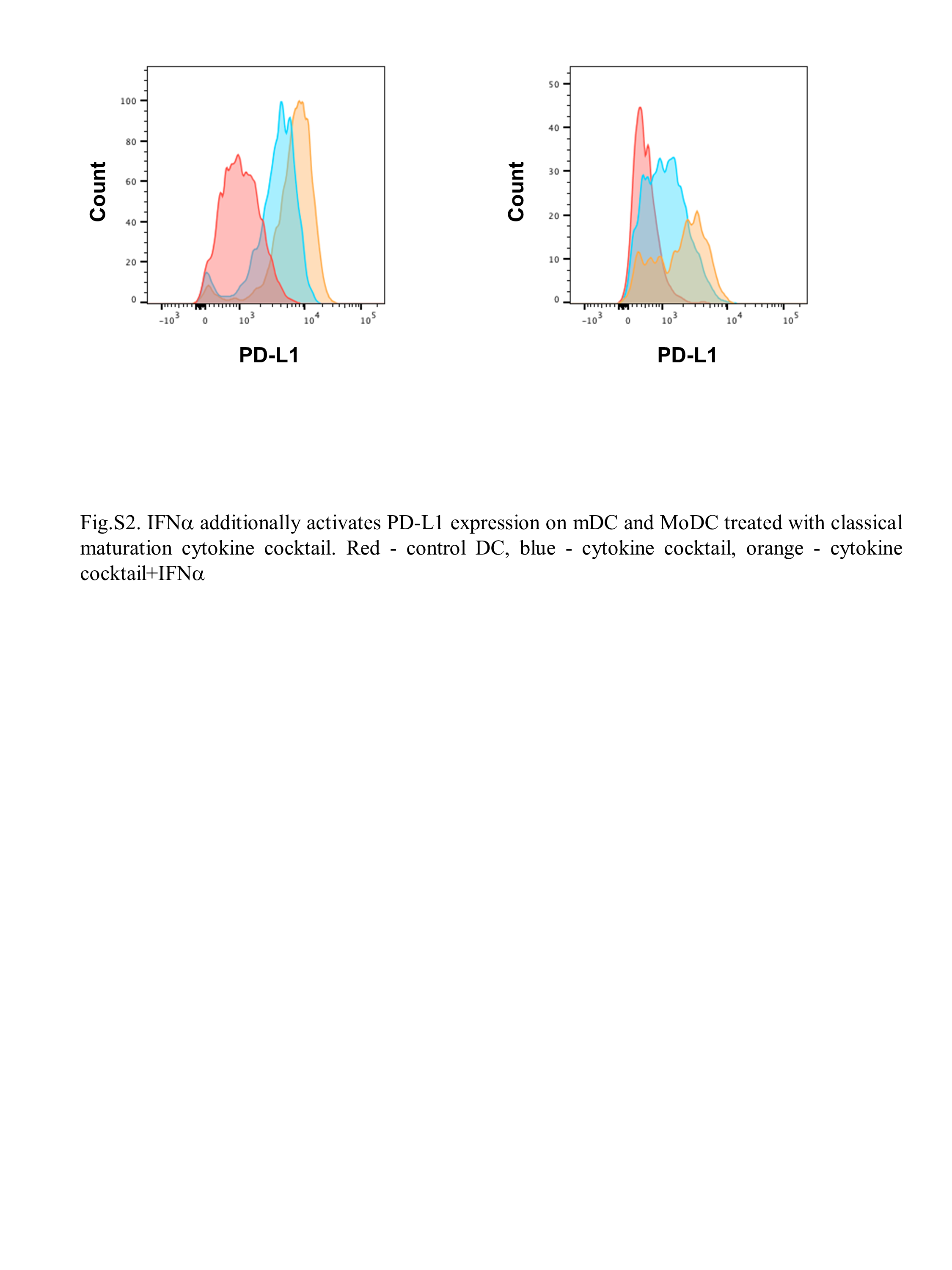

Supplement: Supplementary file 2 [file Image_2.tif]
